# Barriers to rural women’s participation in social insurance for farmers, villagers, and nomads: the case of Iran

**DOI:** 10.3389/fsoc.2024.1433009

**Published:** 2024-11-01

**Authors:** Hamed Ghadermarzi

**Affiliations:** Department of Geography and Rural Planning, Faculty of Geographical Sciences, Kharazmi University, Tehran, Iran

**Keywords:** grounded theory, participation barriers, rural women’s participation, social insurance of farmers, villagers, and nomads, social welfare

## Abstract

**Introduction:**

Since the enactment of the Law of Comprehensive Structure for Social Welfare and Security in Iran, only a small fraction of its target has been accomplished and a significant part of rural women have not been covered by the social insurance service yet. A few studies have been conducted on the social insurance of rural people. However, no study has ever addressed the issue of women with a focus on the theoretical aspects of sociology science, which is the contribution of the present research. Therefore, the present research aimed to explore the barriers to rural women’s participation in social insurance.

**Methods:**

The research adopted a qualitative approach and the grounded theory method. It was conducted among the brokers of social insurance for farmers, villagers, and nomads in Iran. Data were collected through interviews.

**Results and Discussion:**

The results showed that the barriers to women’s participation in social insurance were economic (e.g., women’s economic dependence on the family head), social (e.g., low social trust, low literacy and awareness of rural women, and limitations imposed by religious doctrine), cultural (e.g., limited social communications, limited use of technology, and poor insurance culture), legal (e.g., poor legal support for rural women’s insurance and non-satisfaction of expectations from the fund services), and institutional (e.g., inefficient advertisement methods and poor awareness-raising measures).

## 1 Introduction

In the contemporary world, women, who constitute half of the world population, have a highly important role in developmental programs, and their active participation in all economic, social, cultural, political, environmental, and family contexts guarantees the success of the schemes and programs (Izadi et al., 2019; Jamshidi et al., 2024). So, societies have tried to expand their cooperation in social, economic, cultural, political, and family affairs, increase their share in decision levels and the implementation of development programs (Moradhaseli et al., 2019; Naderi et al., 2024), and create conditions for their flourishing in various aspects by identifying and resolving the barriers to their extensive presence and participation (Shu, 2018; Evangelakaki et al., 2020; Anabestani et al., 2024; Aliabadi et al., 2024).

There are numerous important impediments implicated in women’s low or lack of participation in development programs in different societies (Inneh et al., 2024; Samad et al., 2024), e.g., the social status of women in society, their social awareness (Vu et al., 2024; El Dessouky, 2023), cultural values (such as traditions, customs, values, and social norms), religious and people’s insights, literacy level, cultural pressures (e.g., honor, fidelity, and gratitude to parents) (Sasikumar and Sujatha, 2024), and the dominance of patriarchism (Fan et al., 2021; Giles et al., 2021; Green et al., 2021). Women’s value orientation is a factor that influences their social cooperation. In this regard, family, patterns in the social environment (such as the participation of their members) (Ataei et al., 2021), and awareness of facilities that exist in the living environment are important variables underpinning women’s participation (Gui, 2018; Ataei et al., 2023b; Anbari et al., 2024). Besides social, cultural, and identity issues, women’s economic conditions and features influence the sort and extent of their participation (Liu et al., 2018; Yang, 2018).

Unemployment, elderliness, and incidents are issues that threaten women’s lives more than men’s lives. Women are more vulnerable in rural areas than in urban areas (Wu and Xiao, 2018; Ma and Oshio, 2020; Gholamrezai et al., 2021), so the significance of the social welfare and security system and support insurance system has drawn attention to filling the gaps and covering the likely future risks (Aazami et al., 2019; Moradhaseli et al., 2021). In Iran, given the importance put on the comprehensive system of social welfare and security, Article 29 of the Constitution and Article 96 of the Fourth and Fifth Economic, Social, and Cultural Development Plans have emphasized the implementation of social insurance across rural areas. Social insurance in most countries has been extended to rural areas, targeting both women and men similarly. Social insurance is a major instrument to ensure the security of women’s future and can play a key role in improving their socio-economic status by reducing their poverty and financial dependence. This will happen if women are actively involved in insurance to enjoy this facility.

The Social Insurance Fund of Farmers, Villagers, and Nomads of Iran (SIF) was founded in 2005 and has been active since then. It is voluntary to apply for insurance in SIF, and SIF covers all people in the target community including women and men for its services. People in the age range of 18–50 years can become a member of SIF. Three main services provided for the social insurance of farmers and villagers include elderly insurance (retirement), disability insurance, and life insurance (survivor annuity). This insurance system in Iran is chiefly characterized by the advantage that the government pays 66.7 percent of the insurance premium and the policyholders only pay 33.3 percent, or one-third, of the premium. In other words, the government pays a subsidy of two-thirds of the premium to the policyholders of SIF. Despite this advantage, evidence shows the low participation of women in this fund. According to the latest statistics on SIF, this fund had 1,776,017 members by 2019 out of whom 81 percent (1,439,003 people) were male and the remaining 19 percent (337,016 people) were female. The latest general census of Iran in 2016 shows that the number of rural males and females was 10,630,549 and 10,100,076, respectively. The comparison of these two figures reveals that since the enactment of the Law of Comprehensive Structure for Social Welfare and Security in 2004, only a small fraction of its target has been accomplished and a significant part of rural women are not covered by the social insurance service yet. Similarly, research on poverty shows that women have a greater share of poverty than men, and rural women are more exposed to poverty than urban women (Obadha et al., 2020; Xie et al., 2021; Peng and Ling, 2019; Vaez Roudbari et al., 2024). Although women play important roles in production, family, and diverse activities that they do, they enjoy lower revenue sources. Iran is not an exception and women in its rural areas have lower economic resources despite their participation in agricultural and homemaking activities (Moradhaseli et al., 2017; Ghadermarzi et al., 2022, 2023; Ataei et al., 2023a). Therefore, rural communities in general and rural women in particular are in more urgent need of social insurance and its expansion. The expansion of social insurance across rural communities needs the identification of the reasons and solutions and the proposition of executive courses of action. This, in turn, needs to precisely identify all aspects of the issue and make plans based on scientific findings.

A few studies have been conducted on the social insurance of rural people and yet no study has ever addressed the issue of women with a focus on the theoretical aspects of participation, which is the contribution of the present research. It may be hoped that the results of this study and the recent interest shown by social insurance in the sociological approach is more than a flash in the pan and that a new survey of the subject in a few years will lead to a more positive evaluation of the contribution of sociology. Both sociologists and social insurance practitioners can contribute to this development and benefit from it using the results of this study. The contribution of sociologists should be to undertake more impactful studies, which requires improved knowledge of the user of social insurance and a better definition of the concept of social “insecurity” in order to be able to isolate the effects of social security programs and assess them more accurately in the rural areas. Rural women can be helped to better enjoy the legal capacities of insurance supply by recognizing the barriers to their participation in social insurance and thinking about solutions for tackling them. Finally, by adopting these solutions, SIF, which is the organization in charge of providing insurance in rural areas, can successfully accomplish its mission set by the upstream documents, i.e., the expansion of insurance across all regions. Therefore, the question arises, what are the obstacles to women’s participation in the social insurance of farmers, villagers, and nomads? This research question will be answered in the Results section. The results can be applied in the field of women by SIF officials and planners, the Ministry of Cooperatives, Labor, and Social Welfare, and rural development planners in developing countries.

## 2 Literature review

Ghadermazi et al. (2021) researched the factors affecting the effectiveness of social insurance for farmers, villagers, and nomads in the rural areas of Kurdistan province, Iran. They consider social insurance a part of the social support system for rural people in Iran that pursues such goals as alleviating deprivation and poverty, establishing social justice, and ensuring public welfare. The results show that socioeconomic factors are more influential on rural people’s social insurance than cultural and geographical factors. Varmazyari and Moradi (2017) explored the barriers to the development of social insurance for farmers, villagers, and nomads and proposed five categories of structural, executive, economic, socio-cultural, and motivational barriers. Rezvani and Azizi (2013) studied the challenges of SIF in interviews with some insurance experts in which the barriers to the expansion of social insurance among rural people and nomads were divided into two categories. One category included structural and legal factors, e.g., regulations and lack of proper advertisement, and the other was related to the status of the rural community including distrust in insurance agents, unawareness of insurance, and insurers’ economic inability to pay the insurance premium, especially in less-developed regions.

Shafieezadeh et al. (2013) studied the factors influencing the institutionalization of social insurance in rural areas and categorized them into three groups: cognitive-normative factors, cultural-social factors, and regulatory factors. In a study on the factors affecting farmers’ adoption of social insurance, Sharifi and Hosseini (2009) conclude that the adoption of social insurance is influenced by such factors as the household economic potential, farmers’ lack of trust, household human capital, rural development level, and following others. In a study on the barriers to rural social insurance, classified 11 key concepts as the main problems of rural insurance development among villagers and nomads. They include socio-economic issues, structural-infrastructural issues, motivational-incentive issues, lack of institutional coordination, advertisement and awareness-raising, managerial-executive issues, lack of human resources, professional ethics, and financial-credit issues. Javanmard et al. (2019) discuss that social status, prudence, social trust, social insurance quality, and knowledge and awareness level are effective in rural people’s participation in social insurance.

In a study on the problems of the rural social security system in China and solutions for them, Chen and Yang (2014) reveal that the rural social security system of China has a vital strategy for agricultural development and it has prominent achievements after decades of endeavor. However, there are problems like limited social security coverage due to financial shortages and the government tries to settle them by increasing investment, creating united social security management, emphasizing rural social security, adopting advertisement measures, and completing supervision of the system. In a study in Indonesia, Angelini and Hiros (2004) reported that the weakness of production resources was the main reason for the non-adoption of insurance in the informal economy, and this factor along with the low level of literacy and non-farming skills had limited the expansion of insurance among landless farmers, farmers with limited lands, fishers, marginal users, and women in rural areas. According to this research, the lack of trust between governmental and non-governmental institutions, insurance agents, and farmers was mutual and the reluctance of both parties had led to their low participation. This reluctance as a norm had a negative impact on farmers’ adoption of social insurance. Ramesh (2007) and Yari et al. (2024) specified some barriers to the expansion of social insurance in developing countries. They include villagers’ lack of awareness about rural insurance status and benefits, the limitation of insurance benefits to some specific cases, the focus of insurance agents and firms on urban areas, and the lack of institutional innovation for rural people’s needs, which are different from the urban sector in nature.

Anderson (2001) states that social insurance for farmers is an innovation whose adoption by the target community needs to go through specific steps. He argues that the philosophy of social insurance and its benefits for society requires that the government provides the requirements and support for its adoption by farmers. Akwaowo et al. (2021) and Moradhaseli et al. (2018) argue that social insurance is mainly focused on urban areas and has been neglected in rural areas. Insurance companies adopt various marketing strategies, but their penetration is still lower in rural areas than in urban areas. Bairoliya and Miller (2021) studied the penetration of rural insurance and the reasons for the poor performance of insurance firms and found that the insurance sector was developed in India but it still covered rural areas poorly. The reasons for the low popularity of insurance can be enumerated as unawareness, lack of motivation, and lack of timely payment of indemnity. Bairoliya and Miller (2021), Fan et al. (2021), and Hajrasouliha and Shahgholi Ghahfarokhi (2021) concluded that institutional and legal challenges have hindered the development of social insurance in rural areas in many countries.

The literature review revealed that various factors and components can affect the participation of people in social insurance. These factors may vary with the socio-cultural context of each region and community. Also, most researchers have not provided categories of factors and have examined components separately. If these factors are extracted more comprehensively with different subcategories, a broader perspective for social insurance services will be provided in rural areas.

## 3 Methodology

The research is an applied study in terms of goal and a qualitative study in terms of approach. The statistical population was composed of all brokers of the Social Insurance Fund of Farmers, Villagers, and Nomads (SIF) in 31 provinces of Iran (1,800 brokers). The sample whose size was determined at 317 brokers by Cochran’s formula was taken by the stratified random sampling technique. To distribute the representatives, the proportion of the sample size to the total number of brokers was first determined for each province. Then, a sample was taken from each county considering the geographically proportional distribution of the samples. When the number of representative brokers in a province was fewer than that of its counties, the samples were taken from more populated counties. If the representative brokers of a province outnumber the number of province counties, more than one representative was selected from more populated counties considering the geographical distance of the populated counties from one another.

The research adopted Grounded Theory. Ground Theory refers to what is induced from the study of a phenomenon and represents that phenomenon. In other words, it must be discovered, completed, and proven experimentally by regular collection and analysis of data rooted in the phenomenon. So, data collection and analysis are in a mutual relationship (Bryant, 2020; Khosravi and Amjadian, 2024). The method uses an inductive approach, i.e., going from the specific to the general. With this approach, the researcher shapes a theory by identifying its components. The theory derived from this strategy is closer to real-world facts (Scholes, 2020). The reason for the adoption of this method is that there is no significant research literature available on the barriers to rural women’s participation in social insurance. In addition, to gain an in-depth and all-inclusive understanding of the issue, it seemed necessary to use a method that could provide a comprehensive picture of the participants. So, we employed Grounded Theory as the base method of the research.

The executive steps of Grounded Theory in the research include theoretical sampling, data collection, coding, and data analysis, which was initiated concurrent with the other steps and continued until theoretical saturation. The data were collected through in-depth interviews and using an open-ended questionnaire as the research instrument. The interviews could not be conducted face-to-face due to the COVID-19 pandemic, so they were carried out by telephone. First, the phone numbers of the brokers were collected in each province, and then, the selected brokers were contacted to be invited for telephone interviews. During the phone conversation, the brokers’ responses were jotted carefully.

The data were coded through three steps: open coding, axial coding, and selective coding. In the open coding step, after the main sentences were extracted, the similar meaning components were specified, and then, a code was assigned for each main and core code. In the next step, i.e., concentrated coding, which is the second step of open coding and aims to compare the codes to recognize similar and overlapped codes, the researcher arranged the codes or concepts and integrated similar and common codes within a united category. So, the plenty of data (codes and concepts) were reduced to a certain number of main categories. Before axial coding, the experts and some participants were requested to review the categories and sub-categories derived from open coding to specify if there were any irrational categories. The participants confirmed all categories and sub-categories. In the selective coding step, the relationships of the categories derived from the axial coding step were first specified for which rural women’s participation in social insurance was regarded as the core phenomenon. After the relationships of the identified factors with the core category were specified, the conceptual framework of the research was developed. To ensure reliability, the techniques of triangulation, long-term engagement with data, selection of proper meaning unit, selection of the amount of data required, and the use of quotations and narrations from the jotted text were employed. The research adopted two methods for triangulation. First, it was tried to select people who had different views on the issue at hand. Second, the data were collected by different methods so that they could give their opinions in different conditions. For example, the in-depth interview was conducted individually so that the participants were in different conditions and it was ensured that the environmental conditions or external factors would not influence the results. Finally, the model was provided to several experts to ensure that the results matched the reality.

## 4 Results

**Figure 1:**
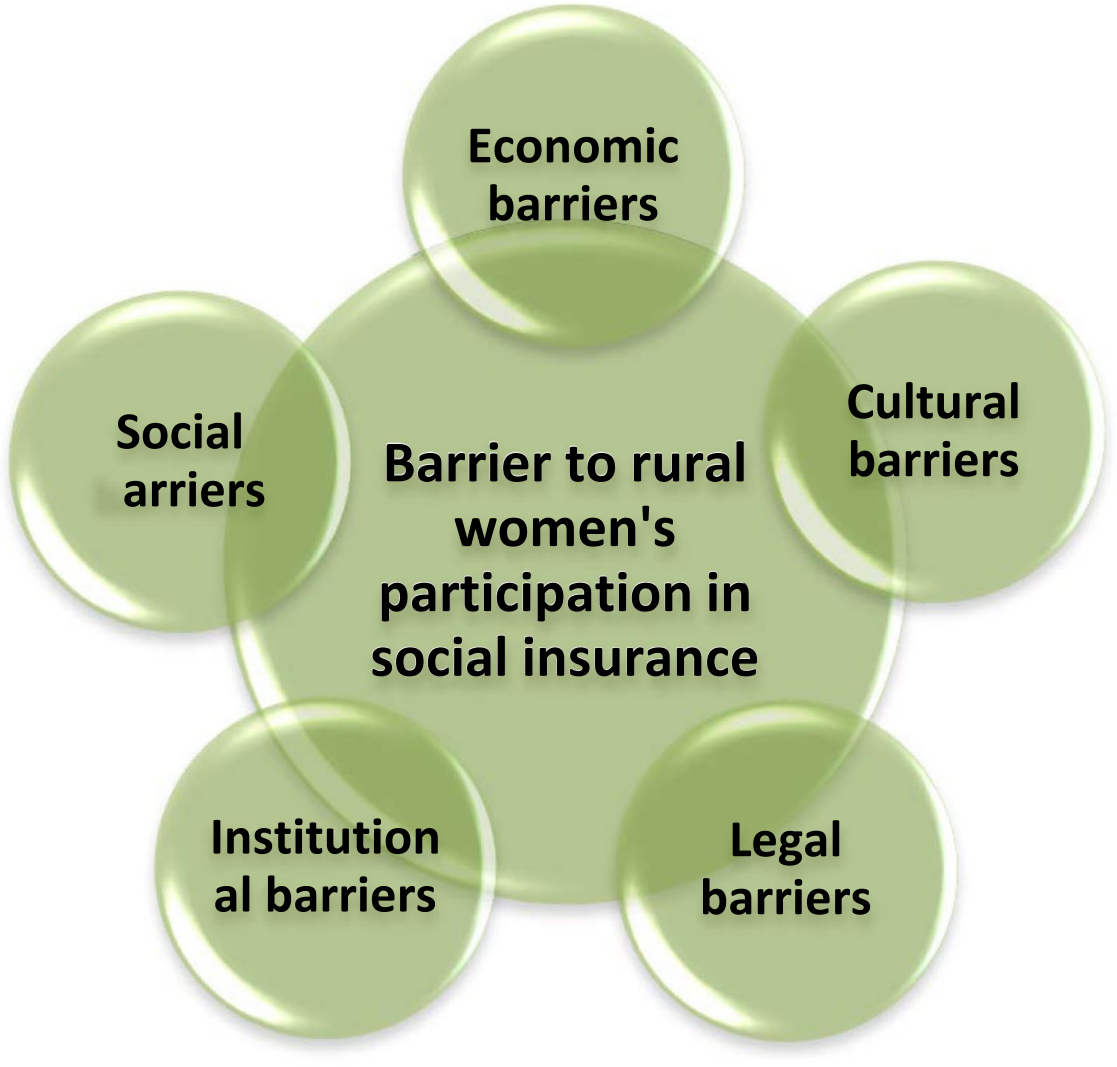
Barriers to rural women’s participation in social insurance.

After the data were subjected to open coding, 118 initial concepts were extracted and 28 key concepts were identified about the research topic. In the open coding step, the full texts of the interviews were checked individually to extract the initial concepts. In the axial coding step, the initial concepts derived from each interview were compared to those of the other interviews, and the key concepts were integrated. In the selective coding step, the key concepts and their dimensions were specified and finally, the core concepts were obtained. They included economic, social, cultural, legal, and institutional concepts. In other words, in response to the research question, *what are the obstacles to women’s participation in the social insurance of farmers, villagers, and nomads*, it should be acknowledged that the barriers to their participation in the social insurance of farmers, villagers, and nomads were categorized within five core concepts (economic, social, cultural, legal, and institutional obstacles) (Figure 1).

### 4.1 Economic barriers

Although rural women have always been a significant part of the workforce and production in rural communities and have been involved in the household’s economic activities, which are mainly related to crop and animal farming, in various forms, their economic activity has been regarded as a part of family labor with no direct wage payment by the family head. This has limited their economic capacity to invest and spend money.

As is evident in Table 1, rural women’s non-participation in social insurance is a function of their economic dependence on the family head (a frequency of 222) and the weak economy of rural families (a frequency of 14). These factors influence one another as rural families in Iran are mainly at the lower income deciles. “Rural women’s lack of job and fixed income,” “weak financial base of rural women,” and “women’s enlistment to be covered by the family head’s insurance” were the main concepts in the category of “women’s economic dependence on the family head.” Also, the three categories of “low income of rural households,” “weakness of the rural household’s financial base to cover the cost of insurance for both husbands and wives,” and “people’s poor economic and livelihood situation in the current year” had the highest impact on the core category of “the poor economy of the rural family” as the frequencies showed.

**Table 1:** The economic barriers to rural women’s participation in social insurance.

Initial concepts	Frequency	Key concepts	Frequency	Core concept
Dependence of insurance adoption on people’s income and livelihood	13	The poor economy of the rural family	140	Economic barriers
Low income of rural households	41			
Widespread poverty in the region and the membership of the majority of people in the Imam Khomeini Relief Foundation	5			
People’s poor economic and livelihood situation in the current year	20			
Weakness of the rural household’s financial base to cover the cost of insurance for both husbands and wives	28			
Lack of a sustainable income	14			
Dependence of rural people’s income on climate change (consecutive droughts)	10			
Non-priority of women’s insurance due to the household’s low income	9			
The weak financial base of rural women	40	Rural women’s economic dependence on the family head	222	
Women’s lack of financial independence	29			
Women’s inability to pay for insurance	13			
Rural women’s lack of jobs and fixed income	51			
The need for the Fund to provide facilities for women	30			
Women’s lack of wages and deprivation from the benefits of their work and economic activity at home	4			
women’s enlistment to be covered by the family head’s insurance	37			
Women’s financial dependence on men	18			

### 4.2 Cultural barriers

The cultural barriers to rural women’s non-participation in social insurance are a function of limited social communications, limited use of technology, poor insurance culture, limited number of exclusive brokers for women, non-acceptance of women’s independence in society, the dominance of patriarchic culture, and the brokers’ poor local knowledge. The most important cultural barrier to rural women’s participation in social insurance is the dominance of patriarchic culture. This key concept is composed of 14 initial concepts, the most important ones being “men’s non-interest in insuring women,” “insurance preference for men according to the ruling culture (patriarchy),” and “acceptance of insurance preference for men by rural women.” Other findings are presented in Table 2.

**Table 2:** The cultural barriers to rural women’s participation in social insurance.

Initial concepts	Frequency	Key concepts	Frequency	Core concept
Women’s limited and insufficient access to smartphones	6	Limited social communications	6	Economic barriers
Ignorance and functional weakness in the use of advertising with virtual world facilities	3	Limited use of technology	3	
Failure to institutionalize insurance culture	29	Poor insurance culture	37	
The non-prevalence of buying insurance for both spouses	8			
Limited acceptance by women due to the masculinity of the brokerage environment	6	Limited number of exclusive brokers for women	12	
The unacceptability of brokers by women	6			
The unpopularity of women’s insurance in this region	1	Non-acceptance of women’s independence in society	6	
Women’s lack of freedom to spend income in different sectors of the rural economy	5			
Acceptance of insurance preference for men by rural women	15	The dominance of patriarchic culture	136	
Men’s non-interest in insuring women	24			
Insurance preference for men according to the ruling culture (patriarchy)	21			
Men’s measures to prevent insurance purchases by women due to the perceived risk of independence-seeking	9			
Parents and spouses not agreeing to purchase insurance	3			
Men’s misconceptions about women’s insurance	8			
Forbidding women to pay for insurance by men	14			
Limited acceptance of local people to accept and work with female brokers	2			
Insurance priority for household heads (men)	14			
Intellectual dependence of women on their husbands	2			
Incorrect prejudice of local and small communities about women’s independent behavior	2			
The low importance and status of women in the target community	5			
Prevalence of cultural and ethnic prejudices in nomadic-rural areas	13			
Low social independence of women and their dependence on husbands	4			
The weakness of brokers in using the ability of the women’s community to advertise insurance for women	8	Brokers’ poor local knowledge	33	
Brokers’ lack of knowledge about the socio-cultural characteristics of society, especially women	7			
The Fund’s not using the ability of local trustees (well-known people, village councils, etc.) to introduce social insurance	8			
Incorrect location of the broker office in terms of environmental security	3			
Low acceptance of most women for insurance	7			

### 4.3 Social barriers

Based on the results, the social barriers to rural women’s participation in social insurance include low social trust, literacy, and awareness of rural women, limitations imposed by religious doctrine, low acceptance of social insurance by rural communities, the dominance of Social Security Insurance in the region, and low social participation of rural women. The key factor of low literacy and awareness was most strongly influenced by “the low level of women’s awareness of social insurance benefits” (a frequency of 24). Likewise, the key factor of low level of social trust was most strongly influenced by the initial factors of “distrust in the Social Insurance Fund of Farmers and Nomads” and “distrust of villagers to government programs,” respectively (Table 3).

**Table 3:** The social barriers to rural women’s participation in social insurance.

Initial concepts	Frequency	Key concepts	Frequency	Core concept
Distrust due to the repeated changes in the rules of the Fund	3	Low level of social trust	40	Social barriers
People’s distrust in the government regarding the payment of insurance premiums	11			
Distrust in the insurance fund	14			
Distrust of villagers to government programs	12			
Poor literacy and illiteracy of rural women	6	Low level of literacy and awareness	46	
Low education and knowledge of women	11			
The low level of women’s awareness of social insurance benefits	24			
Lack of insurance information among rural women	5			
Lack of acceptance of insurance among some Sunni people	7	Limitations imposed by religious doctrine	7	
Disinterest in insurance in rural areas	9	Low acceptance of social insurance by rural communities	9	
The feeling of not needing insurance for the villagers due to the membership of the households in the social security organization	5	The dominance of Social Security Insurance in the region	5	
Indifference toward the future among women’s community	6	Low social participation of rural women	17	
Women’s low level of social communication and their little knowledge of current affairs compared to men	3			
Low level of the legal property right to means of production and so on among women	3			
Lack of self-confidence and the tendency toward isolation in women	5			

### 4.4 Legal barriers

According to Table 4, the legal barriers to women’s non-participation in social insurance are a function of the key concepts of non-satisfaction of expectations from the Fund services (a frequency of 192), non-coverage of the medical sector (a frequency of 178), poor legal support for rural women’s insurance (a frequency of 103), and low diversity of insurance services in the competitive market (a frequency of 35). These challenges in the legal dimension have prevented the Fund from achieving its main goals in developing rural women’s social insurance and have been a source of dissatisfaction. The key factor of non-satisfaction with expectations from the Fund services has been most strongly affected by the concepts of “high retirement age” and “the low amount of the Fund’s pension.” The initial concept of “not having a medical service booklet” was the most important factor for the non-coverage of the medical services. The main factor involved in the poor legal support of rural women’s insurance was “non-payment of pension to survivors of the deceased women.”

**Table 4:** The legal barriers to rural women’s participation in social insurance.

Initial concepts	Frequency	Key concepts	Frequency	Core concept
Contradictory and unequal treatment of the Fund in establishing the pension of insured men and women	3	Poor legal support for rural women’s insurance	103	Legal barriers
Paying more benefits to men than to women	12			
The law of non-payment of two pensions from one fund to one person at the same time (retirement and death)	13			
Non-payment of pension to survivors of the deceased women	52			
Men’s disinterest in paying women’s insurance premiums due to the non-establishment of pensions for women’s survivors	15			
Prioritizing household head by the Insurance Fund of Farmers, Villagers, and Nomads	5			
Inability to register children in insurance documents of young women	3			
Low pension of rural social insurance compared to other insurance	22	Low diversity of insurance services in the competitive market	35	
Women’s preference to be insured in parallel insurance and support organizations with better pensions	13			
Comparison of insurance starting age and social security retirement age by the insured	50	Non-satisfaction of expectations from the Fund services	192	
The negative burden of early retirement pension with minimum pension	8			
Discouragement of women from removing the cost of shrouds and burial	7			
The low amount of the Fund’s pension	34			
High retirement age	66			
Inequality of fund services for women and men	5			
Not providing up-to-date, attractive, and diverse services for women	19			
Non-payment of father’s pension to surviving divorced daughters	3			
Not having a medical service booklet	98	Non-coverage of the medical sector	178	
Lack of connection between social insurance fund and health insurance organization	13			
Non-provision of free medical service booklets and its negative impact on women’s acceptance of insurance	26			
Failure to provide supplementary insurance due to non-coverage of treatment	20			
The need of most rural women for health insurance and the lack of this service in the fund	21			

### 4.5 Institutional barriers

Some weaknesses identified in the research are related to the institutional structure of SIF. As is observed in Table 5, the institutional barriers to rural women’s participation include inefficient advertisement methods, poor awareness-raising measures, non-use of efficient conditions for advertisement, the inefficiency of some brokers, out-of-date training of brokers, weakness in geographically locating a proper place for brokerage offices, negative advertisement against the Fund especially by the Social Security Organization, poor cooperation between rural institutions and brokers, poor administrative system, the vagueness of rural insurance rules for people, continuous changes in rules, and mismanagement. The key factor of poor awareness-raising measures was emphasized as the most essential institutional barrier to rural women’s participation in social insurance. The main concepts constituting this factor include “weakness of advertising in national and local television and radio broadcasts” (with a frequency of 57) and “inadequate awareness-raising measures about insurance” (with a frequency of 45). One another important institutional barrier to rural women’s participation in social insurance was found to be inefficient advertisement methods whose most important constituent concepts included “better advertising of private insurance and social security to attract policyholders” and “ignoring local culture and conditions in the type and extent of advertisement.”

**Table 5:** The institutional barriers to rural women’s participation in social insurance.

Initial concepts	Frequency	Key concepts	Frequency	Core concept
Better advertising of private insurance and social security to attract policyholders	22	Inefficient advertisement methods	63	Institutional barriers
Limited and ineffective awareness-raising measures	11			
Ignoring local culture and conditions in the type and extent of advertisement	15			
Failure to provide uniform guidelines for advertising to brokerages	10			
Lack of focus on introducing social insurance of the Fund and introducing the benefits and services of the Fund to the people	5			
Limited awareness-raising measures and little familiarity of most women with insurance	30	Poor awareness-raising measures	219	
Weakness of advertising in national and local television and radio broadcasts	57			
Lack of visual advertisements in the environment	23			
The low acceptance by women due to the lack of advertisements in the national media	19			
Low use of the conditions created by the COVID-19 pandemic	8			
Inadequate awareness-raising measures about insurance	45			
Ignorance of women about being insured by the Fund	25			
Negative advertising from competitors, especially some private insurance companies	12			
Not using the power of active women in the region to promote insurance, especially for women	3	Non-use of efficient conditions for advertisement	12	
Not using special advertising space to advertise insurance (e.g., national and religious holidays)	3			
Not taking advantage of the conditions created by the COVID-19 pandemic	6			
Lack of brokers’ full command of the Fund rules	10	The inefficiency of some brokers	46	
Brokers’ lack of attention to the fact that women could not visit the brokerage office in person	8			
Brokers’ lack of faith in the Fund’s obligations	3			
Poor communication between the broker and the target community	6			
The broker’s ignorance of the cultural subtleties of rural and nomadic society	5			
Lack of attention to individual and local characteristics in choosing advertisement type	14			
Weakness in the training of brokers	18	Out-of-date training of brokers	27	
The initial preference of the broker and the Fund for insuring household heads (men)	9			
Poor access to brokerage due to inappropriate geographic distribution	9	Weakness in geographically locating a proper place for brokerage offices	9	
Attempts by social security insurance branches to obstruct fund insurance	8	Negative advertisements against the Fund especially by the Social Security Organization	8	
Non-cooperation of organizations related to the Fund in confirming women’s domestic jobs	8	Poor cooperation between rural institutions and brokers	57	
Non-adherence of the Fund’s party organizations to the signed agreements	13			
Non-cooperation of village governors and councils to promote insurance in villages	24			
The Fund’s weak connection with the Administration of Nomadic Affairs in nomadic areas	2			
Lack of cooperation in the Relief Foundation by stopping payment of insurance premiums for clients	7			
The cooperation of the Department of Industry and Mines only with a limited number of brokers in the region	3			
The negative effect of administrative bureaucracy on the establishment of pensions	8	Poor administrative system	8	
The vagueness of the rules regarding the length of years of youth insurance	16	The vagueness of rural insurance rules for people	16	
The vagueness of the rules of the Fund regarding the retirement ceiling	18	Continuous changes in rules	18	
Fund managers’ little understanding of the conditions of villages far from the center	1	Mismanagement	1	

## 5 Discussion

The results showed that legal barriers are among the main obstacles to rural women’s participation in social insurance. Rural women constitute half of the rural population in Iran. However, they are not culturally of the same status as the men, which shows its implications in the form of the non-participation of women in development activities. The culture of rural communities gives limited authority and freedom of communication to women due to the dominance of patriarchic culture. In these cultural conditions, rural women who are used to the ruling culture avoid any behavior that is not within their acquired mental framework. In other words, it can be said that cultural factors penetrate traditions, values, norms, religious beliefs, and insights of people, thereby hindering women’s participation in different affairs, so their participation in social insurance will face problems in Iran’s cultural conditions. The adverse cultural traditions and gender-related cultural beliefs have been mentioned as barriers to participation in Shafiei Sabet et al. (2018), Ataei et al. (2022), and Safari Shali (2008). Similarly, Varmazyari and Moradi (2017) and Dustmohammadloo et al. (2023) reported the poor insurance culture in traditional rural and nomadic communities as the reason for low participation in social insurance.

Some weaknesses identified in this research are related to the institutional structure of SIF. Poor advertisements, the inefficiency of brokers, poor interaction between the Fund and parallel insurance institutions, and poor management created conditions in which rural women’s participation can be expected to be at a low level. Tao (2017), Giles et al. (2021), Rezvani and Azizi (2013), and Afrakhteh et al. (2016) support this finding. They have also stated that structural and institutional barriers can reduce people’s participation in social insurance, making its development difficult in societies.

Presently, equality and justice between women and men have been accepted by people, and planners and policymakers emphasize the need for the participation of both women and men in development programs. As with other human rights, women have the right to be treated as equally as men are in using social insurance, but various social barriers impede them from participating in this insurance. Personal and personality characteristics, the power structure in the family, knowledge and literacy, and social trust are some factors that influence women’s participation in formal activities. The power structure in most rural families of Iran is in favor of men, and women are less informed and naturally less aware of different social affairs than men. Living in these conditions reduces the social trust of women’s community whereas the adoption of social insurance requires awareness, self-confidence, self-belief, and trust in society. Ramesh (2007), Moradhaseli et al. (2023), and Angelini and Hiros (2004) have concluded that the social barriers to women’s participation in social insurance are a function of low social trust and low literacy and awareness.

Although rural women have always been a key part of labor and production in rural communities and have participated in the economic activities of the rural household (which are mostly related to the crop and animal farming sectors) in various forms, their economic activities are regarded as a part of family labor with no wage payment by the family head. This limits the economic capacity of rural women for investment and money spending. Indeed, they can be considered unemployed employed people who are lowly involved in activities that require investment and money spending since they do not receive a direct wage. Participation in social insurance requires paying the premium, which is influenced by the dominance of the economic culture on rural women’s employment. Other researchers (Chen et al., 2017; Javanmard et al., 2019; Green et al., 2021; Moradhaseli et al., 2022) have also regarded economic barriers (e.g., the poor economy of the rural family and women’s economic dependence on the household head) as the main factors that influence women’s participation in social insurance.

The results reveal that legal barriers also hinder rural women’s participation in social insurance. These barriers to the development of the Fund within communities are impediments to their goals and cause dissatisfaction whereas rules and regulations are designed and enacted by planners and policymakers to handle the affairs of communities, organizations, and institutions. If these regulations do not satisfy the needs of the majority, since they have an extensive scope, they will get farther from their goals and become obstacles and challenges. Other authors (Miao et al., 2018; Ataei et al., 2018; Peng and Ling, 2019; Obadha et al., 2020; Ebrahimi et al., 2023) have supported this finding and expressed that awkward and tough regulation will reduce people’s motivation to accept social insurance.

## 6 Conclusion

In the past, family needs were traditionally supplied by family members (Talebi Khameneh et al., 2023; Mansourihanis et al., 2024). Following social and economic reforms and renovations and transformations in living style, the methods of supporting vulnerable people changed, and the social welfare policy was shifted from the traditional style to government-managed support policies. One of the most important social welfare policies in present societies is social insurance, which has been established to support people in old age and during unemployment periods. In other words, governments have adopted measures to cover their urban and rural populations and can supply people’s future by alleviating the implications of old age, poverty, and sickness periods. This is more eminent in rural areas, especially for rural women. Since rural women participate in social insurance to a lesser extent than rural men, we aimed to explore the barriers to rural women’s participation in social insurance in the present work to find out the obstacles to women’s participation, especially in the Social Insurance Fund of Farmers, Villagers, and Nomads (SIF) and to propose solutions for resolving these obstacles, thereby contributing to the realization of social justice, especially gender justice. The results showed that the barriers to rural women’s participation in social insurance are in five broad categories: cultural, institutional, social, economic, and legal barriers. In other words, the results of this study revealed that to improve rural women’s participation in social insurance, the cultural, institutional, social, economic, and legal barriers must be solved by various organizations and people. So, the barriers to rural women’s participation are not limited to only one aspect, but there is a set of factors that impede their participation in insurance. In other words, there must be an all-inclusive view to understand and plan for social insurance development. Each of these factors may be rooted in the macro-policies of the government, social and personal norms, cultures, regional and family conditions, and many more. So, a precise plan is required to tackle each set of these barriers.

According to the results for economic barriers, it is recommended to SIF policymakers to set a premium for women that will allow maximizing their participation considering the economic and income level of non-insured rural women. Cultural barriers, also, show that SIF planners should develop the culture of insurance in the target community by training and thereby contribute to maximizing women’s participation in social insurance by adopting incentive policies for the membership of couples and creating specific benefits and facilities for women who apply for the insurance. Furthermore, since most social barriers are related to rural women’s knowledge and awareness, it is recommended to adopt measures for enhancing their awareness and trust in future policies in order to develop social insurance among rural women. To tackle legal barriers, a revision should be made in the legislation procedure of the Fund and in bylaws and regulations that are enacted with no scientific and experimental support. This shows that to motivate women’s participation in the Fund, it is necessary to revise or eliminate discriminative regulations and develop new regulations as per the needs and opinions of the target community. Finally, to solve the institutional barriers, the higher management of the Fund should lay the ground for implementing sound advertisement programs through available and popular media, create constructive interactions with parallel insurance organizations in rural areas, and cooperate with experienced institutions in rural and nomadic areas optimally.

## Data Availability

The original contributions presented in the study are included in the article/supplementary material, further inquiries can be directed to the corresponding author.
